# Characterisation of community-dwelling older adults with poor appetite

**DOI:** 10.1007/s00394-023-03129-5

**Published:** 2023-03-04

**Authors:** Pia Scheufele, Anja Rappl, Marjolein Visser, Eva Kiesswetter, Dorothee Volkert

**Affiliations:** 1 Institute for Biomedicine of Ageing, Friedrich-Alexander-Universität Erlangen-Nürnberg, Nuremberg, Germany; 2 Institute for Medical Informatics, Biometry and Epidemiology, Friedrich-Alexander-Universität Erlangen-Nürnberg, Erlangen, Germany; 3 Department of Health Sciences, Amsterdam Public Health Research Institute, Vrije Universiteit Amsterdam, Amsterdam, The Netherlands; 4 Institute for Evidence in Medicine, Faculty of Medicine and Medical Center, University of Freiburg, Freiburg, Germany

**Keywords:** Anorexia of ageing, Aged, Chewing problems, Polypharmacy, Depressive symptoms, Weight loss

## Abstract

**Purpose:**

A poor appetite affects up to 27% of community-dwelling older adults in Europe and is an early predictor of malnutrition. Little is known about the factors associated with poor appetite. The present study, therefore, aims to characterise older adults with poor appetite.

**Methods:**

As part of the European JPI project APPETITE, data from 850 participants, aged ≥ 70 years of the Longitudinal Ageing Study Amsterdam (LASA) from 2015/16 were analysed. Appetite during the last week was assessed with a five-point scale and dichotomised into "normal" and "poor". Binary logistic regression was used to examine associations between 25 characteristics from 5 domains—physiological, emotional, cognitive, social, and lifestyle—and appetite. First, domain-specific models were calculated using stepwise backward selection. Second, all variables contributing to poor appetite were combined in a multi-domain model.

**Results:**

The prevalence of self-reported poor appetite was 15.6%. Fourteen parameters from all five single-domain models contributed to poor appetite and were entered into the multi-domain model. Here, female sex (total prevalence: 56.1%, odds ratio: 1.95 [95% confidence interval 1.10–3.44]), self-reported chewing problems (2.4%, 5.69 [1.88–17.20]), any unintended weight loss in the last 6 months (6.7%, 3.07 [1.36–6.94]), polypharmacy defined as ≥ 5 medications in the past 2 weeks (38.4%, 1.87 [1.04–3.39]), and depressive symptoms (Centre for Epidemiologic Studies Depression Scale without appetite item) (1.12 [1.04–1.21]) were associated with an increased likelihood of having poor appetite.

**Conclusion:**

According to this analysis, older people with the characteristics described above are more likely to have a poor appetite.

**Supplementary Information:**

The online version contains supplementary material available at 10.1007/s00394-023-03129-5.

## Introduction

Ageing is often accompanied by a decline in appetite, referred to as anorexia of ageing [1]. Poor appetite is, thus, widespread in the older population and reported in up to 27% of community-dwelling older adults [2–5]. It is associated with a lower energy and protein intake [2], subsequent weight loss [6], and malnutrition [7]. Poor appetite is also related to adverse health outcomes including impaired physical performance, sarcopenia, frailty, disability, and mortality [8]. These adverse consequences reduce the health and quality of life of older adults and place a burden on the healthcare system [9–11].

So far, there is little understanding of the complex network of physiological and non-physiological factors associated with anorexia of ageing. Previous studies reported various cross-sectional associations with variables from five domains: physical, emotional, cognitive, social, and lifestyle. Accordingly, physical factors which can affect appetite include polypharmacy [12, 13], chewing problems [14, 15], functional [16] and sensory impairment [17], poor oral health [16, 18] and chronic pain [19]. Depression is an emotional factor associated with poor appetite in older adults [15, 20–23]. Cognitive factors such as cognitive impairment and dementia [16, 22] as well as the social factor “living alone” [16] and the lifestyle factors “current smoking” [23] and “poor sleep quality” [24] have also been described in relation to a poor appetite in older adults.

These reported associations are based mainly on group comparisons between normal and poor appetite or are restricted to only a limited number of factors and domains. To the best of our knowledge, only one multivariate analysis regarding the association of a broad range of factors from different domains with appetite in community-dwelling older adults is available [18]. As part of the Health, Ageing and Body Composition Study, Lee et al. (2006) investigated the prevalence of self-reported poor appetite and possible cross-sectional associations with 37 factors in 2,169 well-functioning community-dwelling adults aged 70–79 years in the United States. Poor appetite was reported by 12.0% and significantly associated with symptomatic depression, poor self-reported health status, current smoking, chewing problems, visual impairment, weight loss since age 50 and higher plasma concentration of tumour necrosis factor-α (TNF-α).

A careful characterisation of older community-dwelling adults with a poor appetite will help to identify high-risk groups for poor appetite and develop strategies to improve appetite and prevent malnutrition in older adults. Thus, the present study aimed to characterise community-dwelling older adults with poor appetite comprehensively by examining the associations of poor appetite with a range of factors from the physical, emotional, cognitive, social and lifestyle domains.

## Methods

### Study design

For the present analyses, existing data from the Longitudinal Ageing Study Amsterdam (LASA) were used. LASA is a prospective cohort study, based on a nationally representative sample of older adults aged 55 to 85 years, which started in 1992 and is still ongoing. The first cohort sample (*n* = 3805) was randomly selected from municipal registries in three regions in the Netherlands (around Zwolle, Oss, Amsterdam), with an oversampling of the oldest old and men. A second and third cohort were recruited from the same sampling frames 10 and 20 years after the baseline measurements of the first cohort: the second cohort in 2002–2003 (*n* = 1002), the third in 2012–2013 (*n* = 1023). The main objective of LASA is to investigate the determinants and consequences of ageing in community-dwelling Dutch older adults [25]. Since the first measurement wave in 1992, information on different themes (biomaterial, care, physical functioning, emotional functioning, cognitive functioning, social functioning, demographics and work) was collected every 3 years. At each measurement wave, the main interview followed by a medical interview after 4–6 weeks were both conducted in the participant’s home. Participants also completed a self-administered questionnaire between the interviews. Commitment to the study was sustained through continuous contact with the participants as well as invitations to lectures and participation in ancillary studies. Details of the study are described elsewhere [25].

### Study sample

In the latest LASA wave available (wave I: 2015–2016), 2024 older adults (at least 57 years) participated, 13.1% of the original first cohort (1992), 67.0% of the second (2002–2003) and 83.4% of the third cohort (2012–2013). The reasons for drop-out were mainly mortality and to a lesser extent refusal, ineligibility or no contact [25]. Of the 2,024 participants, 1,012 were aged 70 years or older, of whom 850 had appetite information and were included in the present analysis.

### Appetite

Appetite during the last week was assessed during the main interview as part of the Centre for Epidemiologic Studies Depression Scale (CES-D; second item) [26]: “I did not feel like eating, my appetite was poor”. Based on four answer categories (1 “rarely or never”, 2 “some of the time”, 3 “often”, and 4 “most of the time or always”), appetite was dichotomized as normal appetite (1) and poor appetite (2–4) [27].

### Participants’ characteristics

Participants’ characteristics were obtained during the main and medical interviews, except chewing problems, present pain, satisfaction with life, and sleep quality, which were asked in the self-administered questionnaire.

#### Socio-demographic data

Socio-demographic data included age, sex, and education. Education was categorised as high (university, college, or higher vocational education), middle (general secondary education, intermediate and lower vocational education, and general intermediate education), and low (elementary education completed or not completed).

#### Physical domain

Polypharmacy was defined as the use of ≥ 5 medicines in the past 2 weeks [28]. Based on self-reports of chronic diseases, multimorbidity was defined as the presence of ≥ 2 out of 7 chronic diseases (cardiac disease, peripheral atherosclerosis, stroke, diabetes mellitus, obstructive lung disease, arthritis, cancer). Chewing problems affecting food intake were assessed using the question “Due to chewing or swallowing problems, I eat less” (yes/no). Present pain was asked by a subscale of the Nottingham Health Profile, ranging from 5 to 10 [29], with a score of 5 considered as no pain and 6–10 as pain. Hearing problems were assessed by asking the question “Can you hear well enough?” with four possible answers: 1 “yes, without difficulty”, 2 “yes, with some difficulty”, 3 “yes, with much difficulty”, 4 “no, I cannot” and was dichotomized into no hearing problems (1) and hearing problems (2–4). Self-perceived health was assessed with the question ‘How do you rate your health in general?’ with five answer categories 1 “excellent”, 2 “good”, 3 “fair”, 4 “sometimes good/sometimes poor” and 5 “poor”. Categories were summarised into good (1,2) and fair/poor (3–5). Functional limitations were assessed with a validated questionnaire regarding difficulties with performing activities of daily living (ADL: climbing stairs, dressing, rising from a chair, cutting toenails, walking 5 min outside, using public transportation and bathing) [30] and categorised into none, 1 and ≥ 2 limitations. Physical performance was examined using the Short Physical Performance Battery (SPPB) including a chair stand, tandem stand, and walk test. The scores of each test range from 0 to 4, resulting in a sum score of 0 to 12 [31]. Body weight and height were measured and Body Mass Index (BMI) was calculated as weight (kg) divided by height (m) squared. Unintended weight loss in the last 6 months was derived from the question: “Did your weight change in the last 6 months?” (no change/weight gain/weight loss) and combined with the declaration of an unintentional reason for the weight loss. Hospital admission was assessed by asking “Have you been hospitalised in the last six months?” (yes/no).

#### Emotional domain

Anxiety symptoms were measured with the Anxiety subscale of the Hospital Anxiety and Depression Scale (HADS-A) ranging from 0 to 21 [32], and depressive symptoms were measured using the CES-D excluding the appetite item in the total score resulting in a possible score ranging from 0 to 57, with higher scores representing more symptoms [26, 33]. To assess satisfaction with life, the question “How satisfied are you with your life lately?” was asked. The answer categories 1 “very dissatisfied”, 2 “dissatisfied”, 3 “not dissatisfied/satisfied”, 4 “satisfied”, 5 “very satisfied” were summarised into dissatisfied (1,2), not dissatisfied/satisfied (3) and satisfied (4–5).

#### Cognitive domain

Cognitive functioning was measured using the Mini-Mental State Examination (MMSE). The MMSE score ranges from 0 to 30 with scores of ≤ 23 indicating cognitive impairment [34]. Memory complaints were assessed by self-report “Do you have complaints about your memory?” (yes/no).

#### Social domain

Loneliness was assessed by asking the question “I sometimes feel lonely” with the answer categories “no”, “more or less” and “yes”. Household size was asked by the questions “Besides yourself, how many other people are part of your household?” and dichotomised to “living with others” (≥ 1 person/s in the household) and “living alone” (no other person in the household). Further, social network size (count of “the people with whom you are in touch regularly and who are important to you”) was asked. The emotional support received is the mean of the stated support by each person identified in the social network (“you told … about your personal experiences and feelings”; a score of 0: no support to 3: often supported), and the number of confidants identified from the persons in the social network were assessed as well.

#### Lifestyle domain

Current smoking (yes/no) was recorded by asking the question “Do you smoke?”. The consumption of alcohol was assessed according to the Garretsen Indication of present alcohol use: 0 “does not drink”, 1 “light”, 2 “moderate”, 3 “excessive”, 4 “very excessive” [35] and categorised into non-drinker and drinker. Data on physical activity were obtained using the LASA Physical Activity Questionnaire, where physical activity is reported in min/day [36]. Sleep quality was assessed by asking the question “How would you assess the quality of your sleep over the last month?” with the answer categories 1 “very good”, 2 “somewhat good”, 3 “somewhat bad” or 4 “very bad”. The variable was dichotomised into good (1,2) or poor (3,4) sleeping quality.

### Statistical analysis

All variables are described as the median and interquartile range (P25, P75) (continuous variables) or relative frequencies (categorical variables) according to appetite. Differences between participants with normal and poor appetite were analysed using Mann–Whitney *U* test or Chi^2^-test. In addition, the percentage of participants with poor appetite in each variable category was calculated.

In an exploratory approach, associations between participants’ characteristics within each of the five domains and poor appetite were examined by logistic regression analyses. Complete-case analysis was used to cope with missing data. First, for each domain, a binary logistic regression model was established with all potential domain-specific explanatory variables and adjusted for sex, age and education. Backward elimination was employed to find the most parsimonious model based on the likelihood ratio test (variable entry with *p*-value < 0.05, variable removal with *p*-value < 0.10). By nature of this method, only variables that contribute to the outcome remained in the final models. These variables were then combined in one multi-domain model without variable selection. A *p-*value of < 0.05 was considered a statistically significant result. Statistical analysis was performed using SPSS version 26.0 (IBM, Munich, Germany).

## Results

### Prevalence of poor appetite

Of the 850 participants, 6 reported a poor appetite "mostly or always", 27 "occasionally" and 100 "some of the time" in the last week. The overall prevalence of poor appetite was 15.6%.

### Participants’ characteristics

The median age of the sample was 77.4 (73.7, 83.3) years, the median BMI was 26.7 (24.4, 29.5) kg/m^2^; 56.1% were female, 47.9% were multimorbid, 36.9% perceived their health as fair/poor, 38.4% reported memory complaints and 39.6% were living alone.

Participants´ characteristics are presented in Table 1 by appetite. Participants with normal and poor appetite differed significantly in all characteristics except BMI, social network size, emotional support received, number of confidants and physical activity level (Table 1). Poor appetite was particularly common amongst those who reported chewing problems (55.0%), unintentional weight loss in the last 6 months (35.1%) and sometimes feeling lonely (33.9%). In addition, more than one-fourth of participants with reduced cognitive function, hospitalisation in the last 6 months, dissatisfaction with life, poor self-perceived health, poor sleep quality and smoking reported poor appetite.

**Table 1:** Characteristics of older participants of the Longitudinal Ageing Study Amsterdam (2015–2016) by appetite and proportion of participants with poor appetite in each variable category

Characteristic	Normal appetite *n* = 717	Poor appetite *n* = 133	% poor appetite within characteristic	*p*
Covariates				
Age, y ^1^	77.4 (73.6, 82.9) 78.6 ± 6.3	78.7 (75.2, 84.7) 80.1 ± 6.6		0.01
Sex, %				
Female	53.1	72.2	20.1	< 0.01
Male	46.9	27.8	9.9	
Education, %				
Low	19.4	26.3	20.1	0.05
Middle	56.6	57.9	15.9	
High	24.0	15.8	10.9	
Physical factors				
Polypharmacy, %	34.6	60.9	24.8	< 0.01
Missing	6.6	12.8		
Multimorbidity, %	43.9	69.2	22.6	< 0.01
Missing	0.0	0.0		
Chewing problems, %	1.3	8.3	55.0	< 0.01
Missing	10.3	20.3		
Present pain, %	29.6	50.4	24.0	< 0.01
Missing	11.4	17.3		
Hearing problems, %	34.2	40.6	18.1	0.05
Missing	6.8	12.8		
Self-perceived health, %				
Good	68.1	35.3	8.8	< 0.01
Fair/poor	31.8	64.7	27.4	
Missing	0.1	0.0		
Functional limitations (out of 7 ADL), %				
None	34.6	9.8	5.0	< 0.01
One	23.6	19.5	13.3	
Two or more	40.0	66.2	23.5	
Missing	1.8	4.5		
Physical performance score ^1^	7.0 (5.0, 9.0) 7.2 ± 2.6	6.0 (4.0, 8.0) 5.9 ± 2.4		< 0.01
Missing, %	11.3	23.3		
Body Mass Index, kg/m^2 1^	26.7 (24.4, 29.5) 27.3 ± 4.1	26.7 (24.3, 30.3) 27.7 ± 5.3		0.64
Missing, %	7.8	15.0		
Hospitalisation in last 6 months, %	11.6	24.1	27.8	< 0.01
Missing	0.8	1.5		
Any unintended weight loss in last 6 months, %	5.2	15.0	35.1	< 0.01
Missing	11.3	21.1		
Emotional factors				
Anxiety symptoms ^1^	2.0 (0.0, 4.0) 2.6 ± 2.6	4.0 (2.0, 7.5) 5.12 ± 3.7		< 0.01
Missing, %	0.4	0.0		
Depressive symptoms (CES-D score without appetite item) ^1^	12.0 (11.0, 15.0) 12.9 ± 3.6	16.0 (13.0, 19.0) 16.2 ± 4.8		< 0.01
Missing, %	0.6	0.0		
Satisfaction with life %				
Satisfied	87.0	54.9	11.6	< 0.01
Not satisfied/dissatisfied	9.2	22.6	31.3	
Dissatisfied	2.9	6.0	27.6	
Missing	9.9	16.5		
Cognitive factors				
Cognitive impairment (MMSE), %	7.0	15.0	28.6	< 0.01
Missing	0.0	0.0		
Memory complaints, %	36.8	46.6	19.0	0.04
Missing	0.3	0.0		
Social factors				
Sometimes feeling lonely, %				
Yes	10.3	28.6	33.9	< 0.01
No	71.8	51.9	11.8	
More or less	17.0	18.0	16.4	
Missing	0.8	1.5		
Household size, %				
Living alone	37.7	50.4	19.9	< 0.01
Living with others	59.3	42.9	11.8	
Missing	3.1	6.8		
Social network size ^1^	14.0 (9.0, 21.0) 16.3 ± 9.6	13.0 (8.0, 19.5) 14.6 ± 8.5		0.12
Missing, %	7.4	12.0		
Emotional support received ^1^	1.7 (1.1, 2.1) 1.6 ± 0.7	1.6 (1.1, 2.1) 1.6 ± 0.7		0.88
Missing, %	7.8	12.0		
Number of confidants ^1^	2.0 (1.0, 3.0) 2.4 ± 1.8	1.0 (1.0, 3.0) 2.3 ± 2.1		0.15
Missing, %	8.1	15.0		
Lifestyle factors				
Alcohol consumption, %	75.7	60.9	13.0	< 0.01
Missing	6.7	12.8		
Current smoking, %	7.3	13.5	25.7	0.01
Missing	6.6	12.8		
Physical activity, min/d ^1^	125 (28, 188) 137 ± 85	118 (58, 176) 130 ± 100		0.15
Missing, %	0.1	3.0		
Poor sleeping quality, %	14.4	27.8	26.4	< 0.01
Missing	10.6	16.5		

^1^Presented as median and interquartile range (Q1, Q3) and mean ± standard deviation; comparison between participants with normal and poor appetite: for nominal and categorical variables Chi^2^-test and for continuous variables Mann-Whitney *U* test; ADL (activities of daily living), CES-D (Centre for Epidemiologic Studies Depression Scale), MMSE (Mini-Mental State Examination)

### Factors associated with poor appetite

From 25 variables from 5 domains plus 3 covariates, 14 variables and 2 covariates were explanatory of poor appetite and were entered into the final, multi-domain model. From each domain, at least one variable went into the final model. Amongst the covariates, female sex was explanatory of reduced appetite in all single-domain models, age in the emotional and lifestyle domain models, and education in none of the models. Detailed results of the single-domain models can be found in the supplementary table S1.

In the multi-domain model, female sex, polypharmacy, chewing problems, unintended weight loss in the last 6 months, and depressive symptoms were associated with poor appetite (Table 2). The odds of poor appetite were over five times higher in those reporting chewing problems and three times higher in those reporting unintentional weight loss in the last 6 months. Due to missing values, 181 participants had to be excluded from this model. Participants excluded from the final model were significantly more likely to report poor appetite, were significantly older, and reported sometimes feeling lonely, poor subjective health as well as poor cognitive and functional status more often than those included (table S2).

**Table 2:** Associations between characteristics and poor appetite in 669 older participants from the Longitudinal Ageing Study Amsterdam (2015–2016) in a multi-domain model

	Odds Ratio	95% Confidence Interval		*p*
		Lower	Upper	
Age	1.01	0.97	1.06	0.65
Female sex	1.95	1.10	3.44	0.02
Polypharmacy	1.87	1.04	3.39	0.04
Multimorbidity	1.48	0.81	2.69	0.20
Chewing problems	5.69	1.88	17.20	< 0.01
Pain	1.31	0.75	2.30	0.35
Fair/poor self-perceived health	1.75	1.00	3.07	0.05
Any unintended weight loss in last 6 months	3.07	1.36	6.94	0.01
Anxiety symptoms	1.10	0.99	1.22	0.07
Depressive symptoms	1.12	1.04	1.21	< 0.01
Cognitive impairment	1.50	0.58	3.88	0.40
Memory complaints	1.07	0.63	1.83	0.80
Sometimes feeling lonely				
Yes	1.32	0.63	2.79	0.47
More or less	0.89	0.45	1.75	0.73
Alcohol consumption	0.75	0.41	1.36	0.34
Current smoking	1.20	0.50	2.87	0.69
Poor sleeping quality	1.26	0.69	2.32	0.45

Polypharmacy defined as ≥ 5 medicines in last 2 weeks; multimorbidity defined as ≥ 2 chronic illnesses; cognitive impairment defined as Mini-Mental State Examination Score of ≤ 23

## Discussion

In the current cross-sectional analysis of data from Dutch community-dwelling adults aged 70 years and older, out of 25 variables from 5 domains and 3 covariates female sex, the variables polypharmacy, chewing problems, unintended weight loss in the last 6 months, and depressive symptoms were significantly associated with poor appetite.

Poor appetite was present in 15.6% of the participants with the majority reporting this feeling only some of the time and less than 1% mostly or always. This overall prevalence lies in the middle of the range of prevalence rates previously reported in community-dwelling older adults in Europe [15, 37]. Differences between the above-mentioned studies may be due to different population characteristics or different appetite assessment methods.

In group difference tests, 22 of 28 variables from all domains were associated with poor appetite (Table 1). Interestingly, BMI was not associated with poor appetite. An association between poor appetite and lower BMI was described in community-dwelling older adults which disappeared, however, after age adjustment [38]. Lee et al. reported a significant group difference, but no association in the multivariate analysis [18]. Notably, poor appetite does not necessarily lead to reduced food intake and consequently to a lower BMI. Similarly, physical activity levels did not differ between participants with normal and poor appetite although one might assume a mutual influence, and an association was observed in previous studies [18, 23, 38]. This might be partly explained by the complexity of assessing physical activity. Regarding social factors, social network characteristics did not differ between the groups with poor and normal appetite, whilst sometimes feeling lonely as well as living alone were associated with poor appetite. Thus, the subjective feeling of loneliness and lack of company whilst eating seem more relevant to appetite than the size of the social network.

In the multi-domain model, as in all single-domain models, female sex was significantly associated with poor appetite, which is in line with previous research in community-dwelling older adults [18, 22, 37] and institutionalised older adults [39]. The sex difference could not be explained by older age or higher prevalence of depression in women in our study. In contrast to our results, in a mixed population from different settings, males more often reported having a poor appetite [15]. The association of polypharmacy and poor appetite in the present study has not been reported before in community-dwelling older adults. Poor appetite is a known side effect of some drugs [39] that could be magnified by polypharmacy [40]. In contrast, Landi et al. reported no significant association of poor appetite with the number of drugs taken by nursing home residents aged 65+ years [41]. Future research needs to further examine the possible link between polypharmacy and poor appetite in community-dwelling older adults. Participants with chewing problems were most likely to have poor appetite (Table 2). This is in line with results from previous studies reporting associations between chewing pain [15], chewing efficiency [18] as well as chewing capacity [14] and poor appetite; however, the low prevalence of chewing problems in our sample must be taken into account. More important, when interpreting this association, the assessment method and the consequent possible overlap of the variable with poor appetite should be kept in mind. Chewing problems were assessed via the statement “Due to chewing or swallowing problems, I eat less” and are, therefore, not assessed independently, but in the context of eating less. The resulting possible overlap with the question on poor appetite may lead to an overestimation of the association between the two variables. Unintended weight loss in the last 6 months was also associated with poor appetite. Lee et al. reported an association between weight loss since the age of 50 with poor appetite [18]. Further studies are needed to better understand both the link and the direction of the association. Depressive symptoms were also associated with poor appetite, which is plausible since poor appetite is a well-known concomitant of depression and is one of the 20 criteria to assess depressive symptoms according to the CES-D. In the present analysis, the appetite item was removed from the CES-D score to avoid overlap and methodological interrelations. In the group with poor appetite, without the appetite item included in the scale, the median score was exactly the cut-point of 16, reflecting individuals at risk for clinical depression. The association of depression and depressive symptoms with poor appetite has been reported in several previous studies in community-dwelling [15, 18, 22, 23] and institutionalised [20, 21, 41] older adults.

Interestingly, age, multimorbidity, present pain, fair/poor self-perceived health, anxiety symptoms, reduced cognitive function and memory complaints, sometimes feeling lonely, alcohol consumption, smoking and poor sleep quality were all significantly associated with poor appetite in the single-domain models, but no longer in the final multi-domain model, despite plausible theoretical connections and some evidence of associations with poor appetite in previous studies [18, 19, 22, 24, 42, 43]. Data on anxiety in the context of anorexia of ageing are largely lacking. In the present study, the multivariate analysis showed a borderline significant association. In the group comparison and the single-domain model, higher anxiety symptoms were significantly associated with poor appetite. Lee et al. also observed a significant group difference in anxiety; however, an association was not observed in the multivariate analysis [18]. According to previous research, community-dwelling older adults of a higher age are more likely to report poor appetite [18, 22, 23]. A meta-analysis from Gienzaar et al. of the associations between ageing and decreases in appetite and energy intake in 2016 reported that appetite is reduced in healthy older adults compared with younger adults [43]. However, this association was neither evident in the regression analysis of the final model of the present study nor the regression analysis of Lee et al. [18]. Since these were restricted to people aged at least 70 years, it could be that the association diminishes or disappears at older ages or that the association disappeared due to the adjustment for a variety of age-related variables which might be more relevant than age per se. Cognitive, social and lifestyle factors were not associated with poor appetite in the final multivariate analysis of the present study (Table 2), and therefore seem to be less relevant according to our analyses. Regarding cognitive function, however, there is evidence from group comparisons [15, 22, 23] and one multivariate analysis [42] suggesting a link between reduced cognitive function and poor appetite. Possibly, this association may be dependent on the severity of cognitive impairments. Loneliness might adversely affect appetite via depressed mood [44]. In the present study, sometimes feeling lonely was significantly associated with poor appetite in the group difference test and the social domain model, but no longer associated in the multi-domain model. Previous studies observed that older adults who always eat alone [18] or live alone [45] are more likely to report poor appetite. Regarding lifestyle factors, consistent with the results of the present analysis, no significant association between alcohol consumption and poor appetite has been found so far [2, 23]. A possible link between sleep quality and appetite in older adults has only been examined in a study from Japan [24].

Overall, it can be summarised that despite different variables, domains and statistical approaches, our results are largely consistent with the multivariable analysis from Lee et al. [18]: female sex, weight loss, fair/poor self-perceived health and chewing problems from the physical domain, as well as depression from the emotional domain, were significantly associated with poor appetite in both analyses. In addition, we found polypharmacy associated with poor appetite and Lee et al. smoking, TNF-α, and visual impairment, the latter two variables unfortunately not being available in our dataset.

### Strengths and limitations

The analysis conducted had notable strengths. A large number of variables from important areas of life were assessed in a large sample of European older adults. Five different domains were analysed separately to allow the identification of relevant domain-specific factors. The analysis also evaluated more recent data compared to the only comparable multivariate analysis from Lee et al., 2006 [18].

Several potential limitations of this study should, however, also be addressed. First, in this secondary data analysis, available information was restricted to a limited number of variables with given answer categories that were not tailored to our research question. Further, due to missing values and the nature of the complete case analysis, we were not able to analyse the full sample of 850 participants in the final logistic regression model and a selection bias must be assumed. The subpopulation excluded from the single-domain models as well as the final model due to missing values was significantly older, and more functionally impaired, and thus our sample was not representative of all community-dwelling older adults. Our prevalence rate of poor appetite and the observed associations may have been underestimated. Further, this cross-sectional analysis does not allow any conclusions about intra-individual variations or the causality of the associations. Lastly, in this analysis, appetite was assessed with a single, self-reported and oriented question with only four answer categories, addressing the frequency of poor appetite in the last week. In older adults, Likert Scale or Visual Analogue Scale methods are mostly used [46]. Unfortunately, data from these methods were not included in the LASA dataset. It is up to future studies to assess appetite more comprehensively, e.g. via SNAQ and potentially add objective measures (appetite-related hormones, ad-libitum test meal). Comparisons of the present study with other studies on appetite in older adults must be interpreted cautiously due to the different ways in which appetite is assessed.

## Conclusion

The prevalence of self-reported poor appetite in the present analysis was 15.6%. The multivariate analysis of 28 variables from several domains contributes to the limited knowledge in the field of poor appetite in community-dwelling older adults in Europe. Older persons with a poor appetite were characterised by female sex, polypharmacy, chewing problems, unintentional weight loss in the last 6 months and depressive symptoms. The results highlight the complexity of appetite regulation in older people, where factors from different domains are involved.

In clinical practice, the nutritional status of patients with at least one of the above-mentioned characteristics should be closely monitored as these characteristics may indicate a poor appetite, and thus an increased risk of malnutrition.

## Supplementary Information

Below is the link to the electronic supplementary material.

Supplementary file1 (PDF 338 kb)

## Data Availability

Data from the Longitudinal Ageing Study Amsterdam (LASA) are available for use for specific research questions, provided that an agreement is made up. Research proposals should be submitted to the LASA Steering Group, using a standard analysis proposal form that can be obtained from the LASA website: www.lasa-vu.nl. Files with data published in this publication are freely available for replication purposes and can be obtained using the same analysis proposal form. The LASA Steering Group will review all requests for data to ensure that proposals for the use of LASA data do not violate privacy regulations and are in keeping with informed consent that is provided by all LASA participants.

## Abbreviations

ADL: Activities of daily living
BMI: Body Mass Index
CES-D: Centre for Epidemiologic Studies Depression Scale
HADS-A: Hospital Anxiety and Depression Scale
LASA: Longitudinal Ageing Study Amsterdam
MMSE: Mini-Mental State Examination
SNAQ: Simplified Nutritional Appetite Questionnaire
SPPB: Short Physical Performance Battery Test (SPPB)
TNF-α: Tumour Necrosis Factor alpha
