# Supplementary material for: Characterisation of community-dwelling older adults with poor appetite
Source: Eur J Nutr. 2023 Mar 4;62(5):1991–2000. doi: 10.1007/s00394-023-03129-5 (PMC10349734; doi:10.1007/s00394-023-03129-5)
Supplement: Supplementary file 1 — Supplementary file1 (PDF 338 kb) [file 394_2023_3129_MOESM1_ESM.pdf]

Supplemlentary information

Characterisation of community-dwelling older adults with poor appetite

Pia Scheufele; Anja Rappl; Marjolein Visser; Eva Kiesswetter; Dorothee Volkert

Table S1 – Associations between characteristics and poor appetite in older participants from the Longitudinal Ageing Study Amsterdam (2015-2016) in five single-domain models

	Odds Ratio	95% Confidence Interval		*p*
		Lower	Upper	
**Physical domain** (*n*=662)				
Female sex	1.91	1.10	3.32	0.02
Polypharmacy	1.92	1.08	3.43	0.03
Multimorbidity	1.64	0.91	2.98	0.10
Chewing problems	6.75	2.16	21.06	<0.01
Pain	1.71	0.98	2.96	0.06
Fair/poor self-perceived health	2.20	1.26	3.82	0.01
Any unintended weight loss in last 6 months	3.33	1.48	7.46	<0.01
**Emotional domain** (*n*=754)				
Age	1.04	1.00	1.07	0.04
Female sex	1.97	1.22	3.19	0.01
Anxiety symptoms	1.18	1.09	1.28	<0.01
Depressive symptoms	1.13	1.06	1.21	<0.01
**Cognitive domain** (*n*=848)				
Female sex	2.27	1.50	3.41	<0.01
Cognitive impairment	2.08	1.18	3.67	0.01
Memory complaints	1.45	0.99	2.13	0.06
**Social domain** (*n*=751)				
Female sex	2.04	1.29	3.22	<0.01
Sometimes feeling lonely (yes)	3.41	2.01	5.78	<0.01
Sometimes feeling lonely (more or less)	1.61	0.94	2.76	0.09
**Lifestyle domain** (*n*=727)				
Age	1.04	1.01	1.08	0.02
Female sex	2.01	1.25	3.24	<0.01
Current smoking	2.02	1.02	4.02	0.05
Alcohol consumption	0.59	0.37	0.96	0.03
Poor sleeping quality	2.36	1.47	3.79	<0.01

Odds Ratio adjusted for age, sex and education; variable selection using backwards selection; polypharmacy defined as ≥ 5 medicines in last 2 weeks; multimorbidity defined as ≥ 2 chronic illnesses; cognitive impairment defined as Mini-Mental State Examination Score of ≤ 23

Table S2 – Characteristics of participants included in the multi-domain model and excluded from the multi-domain model

Characteristic	Included<br>*n* = 669	Excluded<br>*n* = 181	***p***
**Covariates**			
**Age**, y [1]	76.7 (73.5, 81.5)<br>78.0 ± 5.8	82.5 (76.3, 87.4)<br>82.2 ± 7.3	<0.01
**Sex**, %			0.06
Female	54.7	61.3	
Male	45.3	38.7	
**Education**, %			0.04
Low	18.7	27.1	
Middle	56.5	58.0	
High	24.8	14.9	
**Poor appetite**, %	13.6	23.2	<0.01
**Physical factors**			
**Polypharmacy**, %	38.9	28.2	<0.01
Missing	0.0	35.4	
**Multimorbidity**, %	46.9	51.4	0.05
**Chewing problems**, %	2.8	0.6	<0.01
Missing	0.0	55.8	
**Present pain**, %	36.9	17.7	<0.01
Missing	0.0	58.0	
**Hearing problems**, %	36.6	29.8	<0.01
Missing	0.0	36.5	
**Self-perceived health,** %			<0.01
Good	66.1	51.4	
Fair/poor	33.9	48.1	
Missing	0.0	0.6	
**Functional limitations (out of 7 ADL),** %			<0.01
None	34.1	18.2	
One	25.6	13.3	
Two or more	38.7	64.1	
Missing	0.0	4.4	
**Physical performance score** [1]	7.0 (5.0, 9.0)<br>7.2 ± 2.6	6.0 (3.0, 9.0)<br>6.2 ± 2.8	<0.01
Missing, %	6.9	36.5	
**Body Mass Index**, kg/m² [1]	26.7 (24.4, 29.4)<br>27.3 ± 4.2	26.7 (24.2, 30.3)<br>27.7 ± 4.8	<0.01
Missing, %	0.7	39.2	
**Hospitalisation in last 6 months**, %	12.1	18.8	<0.01
Missing	0.0	3.9	
**Any unintended weight loss in last 6 months**, %	7.2	5.0	<0.01
Missing	0.0	60.2	
**Emotional factors**			
**Anxiety score** [1]	2.0 (1.0, 4.5)<br>2.9 ± 2.9	3.0 (1.0, 5.0)<br>3.5 ± 3.3	0.02
Missing, %	0.0	1.7	

**Depressive symptoms (CES-D score without appetite item)** [1]	13.0 (11.0, 15,0)<br>13.3 ± 3.8	13.0 (11.0, 16.0)<br>14.0 ± 4.6	0.18
Missing, %	0.0	2.2	
**Satisfaction with life: lately** %			<0.01
Satisfied	84.0	38.7	
Dissatisfied	3.4	3.3	
Not satisfied/dissatisfied	12.1	8.3	
Missing	0.4	49.7	
**Cognitive factors**			
**Cognitive impairment (MMSE), %**	5.1	19.9	<0.01
**Memory complaints, %**	38.3	38.7	0.35
Missing	0.0	1.1	
**Social factors**			
**Sometimes feeling lonely, %**			<0.01
Yes	10.5	23.2	
No	72.2	55.8	
More or less	17.3	16.6	
Missing	0.0	4.4	
**Household composition, %**			<0.01
Single	37.5	47.5	
With others	61.1	40.3	
Missing	1.3	12.2	
**Social network size** [1]	14.0 (9.0, 21.0)<br>16.3 ± 9.5	12.0 (8.5, 20.0)<br>14.6 ± 8.9	<0.01
Missing, %	3.1	26.5	
**Mean emotional support received** [1]	2.0 (1.0, 3.0)<br>1.6 ± 0.7	2.0 (1.0, 3.0)<br>1.7 ± 0.7	<0.01
Missing, %	3.6	26.5	
**Number of confidants** [1]	1.7 (1.1, 2.1)<br>2.4 ± 1.9	1.8 (1.2, 2.2)<br>2.2 ± 1.9	<0.01
Missing, %	4.2	27.6	
**Lifestyle factors**			
**Current moking, %**	9.1	5.0	<0.01
Missing	0.0	35.4	
**Alcohol consumption, %**	81.8	42.5	<0.01
Missing	0.0	35.9	
**Physical activity,** minutes/day [1]	128 (76, 189)<br>139 ± 83	108 (50, 180)<br>122 ± 98	<0.01
Missing, %	0.3	1.7	
**Poor sleeping quality, %**	19.1	6.6	0.37
Missing	0.0	54.1	

[1] Presented as median and interquartile range (Q1, Q3) and mean ± standard deviation; comparison between participants with normal and poor appetite: for nominal and categorical variables Chi²-test and for continuous variables Mann-Whitney *U* test; ADL (activities of daily living), CES-D (Centre for Epidemiologic Studies Depression Scale), MMSE (Mini-Mental State Examination)
